# Screening of the transcriptional regulatory regions of vascular endothelial growth factor receptor 2 (VEGFR2) in amyotrophic lateral sclerosis

**DOI:** 10.1186/1471-2350-8-23

**Published:** 2007-04-24

**Authors:** Alice Brockington, Beatrijs Wokke, Hannah Nixon, Judith Hartley, Pamela J Shaw

**Affiliations:** 1 Academic Neurology Unit, University of Sheffield, E Floor, Medical School, Beech Hill Road, Sheffield S10 2RX, UK; 2 Leiden University Medical Centre, Albinusdreef 2, 2333 ZA Leiden, The Netherlands

## Abstract

**Background:**

Vascular endothelial growth factor (VEGF) has neurotrophic activity which is mediated by its main agonist receptor, VEGFR2. Dysregulation of VEGF causes motor neurone degeneration in a mouse model of amyotrophic lateral sclerosis (ALS), and expression of VEGFR2 is reduced in motor neurones and spinal cord of patients with ALS.

**Methods:**

We have screened the promoter region and 4 exonic regions of functional significance of the VEGFR2 gene in a UK population of patients with ALS, for mutations and polymorphisms that may affect expression or function of this VEGF receptor.

**Results:**

No mutations were identified in the VEGFR2 gene. We found no association between polymorphisms in the regulatory regions of the VEGFR2 gene and ALS.

**Conclusion:**

Mechanisms other than genetic variation may downregulate expression or function of the VEGFR2 receptor in patients with ALS.

## Background

Amyotrophic lateral sclerosis (ALS) is a fatal neurodegenerative disorder, in which loss of motor neurones in the spinal cord, brainstem and cerebral cortex causes progressive paralysis. Ten percent of cases are familial, and one fifth of these are caused by a mutation in the gene encoding superoxide dismutase (SOD1). In the majority of familial and sporadic cases of ALS, the cause of selective loss of motor neurones is unknown, but there is growing evidence of the importance of dysregulation of vascular endothelial growth factor (VEGF) as a factor in motor neurone degeneration.

VEGF is an endothelial cell mitogen essential for angiogenesis, and upregulated by hypoxia. In 2001, Oosthuyse *et al *found that deletion of the hypoxia response element of the VEGF gene in mice caused motor neurone degeneration with clinical and pathological features similar to ALS [1]. VEGF was subsequently shown to act as a neurotrophic factor *in vitro *and *in vivo *[1,2]. VEGFR2 (also known as KDR) is the major agonist receptor of VEGF, and has been shown to mediate its neuroprotective effects *in vitro *[1]. Neuronal overexpression of VEGFR2 in SOD1 transgenic mice delays the onset of motor impairment, and prolongs survival [3]. A recent immunohistochemical study showed that the expression of both VEGF and VEGFR2 was downregulated on anterior horn cells, and VEGFR2 immunostaining in the neuropil was decreased in the spinal cord in patients with ALS, compared to normal controls [4].

The role of VEGFR2 in the mediation of the neurotrophic effects of VEGF, and its reduced expression in neural tissue of ALS patients identify it as an important candidate gene in ALS. We have sequenced the 5' UTR and promoter region of the VEGFR2 gene in patients with ALS, to determine whether the observed downregulation of VEGFR2 expression in ALS patients is related to the segregation of certain alleles at polymorphic sites within the regulatory regions of the VEGFR2 in the ALS population. We have screened the regulatory regions and 4 exons of functional significance in the VEGFR2 gene for mutations in ALS patients, which are not present in control populations. These 4 exons were identified for screening both on the basis of their functional significance [5], and the presence of previously published polymorphisms within the coding regions [6]

## Methods

### Population screened

Exons 7, 18, 21 and 27 were sequenced in 100 ALS patients, and exon 7 in 100 unrelated neurologically normal controls. The regulatory regions were sequenced in 301 ALS patients and 239 unrelated neurologically normal controls from the north of England. Patients had a diagnosis of definite or probable ALS by the El Escorial criteria. We included patients with the progressive muscular atrophy (PMA), primary lateral sclerosis (PLS) and progressive bulbar palsy (PBP) clinical variants. Approval for the use of DNA samples was obtained from the local ethics committee.

### DNA extraction and PCR reactions

Genomic DNA was extracted from blood, using the Nucleon BACC3 extraction kit (Tepnel UK), or snap frozen human motor cortex using the Nucleon Soft Tissue Kit (Tepnel UK). PCR was performed in a 25 μl volume containing 100 ng of genomic DNA, 10 pmol of each primer, and 12.5 μl of 2× ReddyMix™ PCR Master Mix (ABgene). 5% DMSO was added to the reaction mix to amplify the promoter region. After an initial denaturing step of 95°C for 5 mins, samples were amplified in 35 cycles of 95°C 30 s, annealing temperature 30 s, 72°C 45 s, followed by a final incubation of 72°C for 10 minutes. Primer pairs were obtained from MWG Biotech. (Table 1 details primer pairs and annealing temperatures)

**Table 1:** Primers used to amplify regulatory and exonic regions of the VEGFR2 gene, and polymorphisms present.

	Polymorphisms	Amino acid change	SNP reference	Forward primer	Reverse primer	Annealing temperature
Promoter	-305 C/T	n/a	Novel	GCGACCCGGCATACTTG	AGGCGGCCCGGGTCTCCAC	55
	-263 C/T		Novel			
	-123 C/G		Rs 10027862			
	-65 C/T		Rs 9994560			
	-57 C/T		Novel			
	-17 A/T		Rs 6824124			
5' UTR	+31 G/A		Rs 7667298			
Exon 7	+1192 G/A	Val to Ile	Rs 2305948	CTGGTGTCCCTGTTTTTAGCAT	GTATATCAGCACATCTCATCTTTA	50
Exon 18	+2846 T/A	Val to Glu	Rs 1139776	TAATTAAGCCAGGACAAAGGAGTA	CTAGTAGGCCCACATAAGCACA	50
Exon 21	+3157 T/C	Val to Ile	Rs 13129474	TTCAATTATCTCCATGGTTTACTA	TCACTTTTGGGGAGACAGAAT	46
Exon 27	+3931 C/G	Pro to Ala	Rs 11540507	CACCCTATGTAGCCAAGAAGTC	CTACAGATGGGAGGAAGCACAC	53

SNP reference obtained from ensembl database for known polymorphisms; Novel polymorphisms are those identified in this study. Primers are listed 5' to 3'

### Mutation screening and detection of polymorphisms

PCR products of DNA samples were sequenced directly with an ABI 3730 DNA analyser, using the Big Dye^® ^Terminator Cycle Sequencing Kit, according to manufacturer's instructions. VEGFR2 sequence was taken from ENSCAFG00000002079 on the ensembl database [6], which was used to determine sites of known polymorphisms. Numbering of nucleotides is relative to the translation start site. Polymorphism -57C/T was confirmed using restriction digest with NlaIV. Reaction conditions were: 5 μl PCR product, 1 unit of NlaIV (New England Biolabs), 1×NEB buffer 4, 100 μg/ml BSA in a total volume 10 μl; incubated at 37°C for 1 hour.

### Statistics

Allele frequencies were analysed using Chi-Square test.

## Results

All patients and controls were from Caucasian backgrounds. 58% of patients were male, and the mean age was 61.8. 8.6% of patients had one of the clinical variants of ALS (5.6% PMA, 2% PLS and 1% PBP). In the control population, 56% were male, and the mean age was 56.6.

Exons 7, 18, 21 and 27 of the VEGFR2 gene were screened for mutations or polymorphisms in 100 ALS patients. Exon 7 lies within the VEGF binding domain and exons 18 and 21 within the protein kinase domain. The function of exon 27 is unknown, but it is a region that is highly conserved across species. No mutations were identified in the exons or intron/exon boundaries of exons 7, 18, 21 or 27. The previously described polymorphisms in exons 18, 21 and 27 were not present in this population of ALS patients. The A allele at position +1192 in exon 7 was present at a frequency of 0.06 in both ALS and control populations (Table 2), consistent with its published frequency [6].

**Table 2:** Frequencies of polymorphisms in the regulatory regions and exon 7 in ALS and control populations

Polymorphism	Allele	ALS patients	Controls	Chi squared
		Allele Frequency	Allele Frequency	

-305 C/T	C	262 (0.44)	179 (0.43)	
	T	340 (0.56)	239 (0.57)	0.838
-263 C/T	C	450 (0.75)	303 (0.72)	
	T	152 (0.25)	115 (0.28)	0.381
-65 C/T	C	293 (0.49)	309 (0.50)	
	T	210 (0.51)	208 (0.50)	0.621
+31 G/A	G	325 (0.54)	277 (0.56)	
	A	232 (0.46)	186 (0.44)	0.641
+1192 G/A	G	182 (0.94)	180 (0.94)	
	A	12 (0.06)	12 (0.06)	0.979

**Figure 1:**
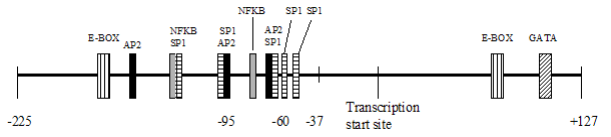
Diagrammatic representation of the regulatory regions of the VEGFR2 gene from -225 to +127, showing consensus regions for transcription factor binding sites.

**Figure 2:**
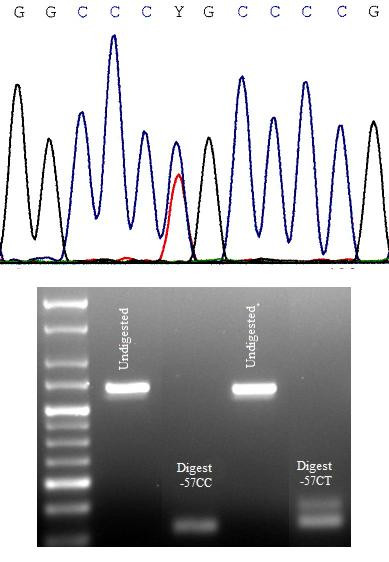
a) Sequence data showing C/T heterozygosity at position -57; C = cytosine, G = guanine, Y = pyrimidine b) Digest of PCR products of regulatory region amplification with enzyme NlaIV, showing a restriction fragment length polymorphism in case B, due to the presence of allele -57T. NlaIV cleaves the PCR product in the presence of the C allele, but not the T allele. Lane one contains the hyperladder marker of molecular weight.

The VEGFR2 5' UTR and promoter region was sequenced from -315 to +302 relative to the transcription start site, which includes the core promoter region of the VEGFR2 gene (Figure 1) [7], in 301 ALS patients and 239 unrelated neurologically normal controls. Five polymorphisms were identified in these regulatory regions, of which three were novel (305 C/T, -263 C/T, and -57 C/T). Previously described polymorphisms [6] -123C/G and -17A/T were not seen. There was no difference in frequency of polymorphisms -305 C/T, -263 C/T, -65 C/T and +31 A/G in the ALS population, relative to the control population (Table 2). The novel polymorphism -57 C/T is located in a consensus sequence for binding SP1, the major transcriptional regulator of VEGFR2 [8,9], and was found in one patient and one control case (Figure 2)

## Discussion

The VEGF signalling pathway has an important role in the maintenance of neuronal survival, and dysregulation of this pathway leads to motor neurone death. A meta-analysis of pooled European populations demonstrated that two haplotypes of functional polymorphisms in the 5' UTR of the VEGF gene conferred a 1.8 fold greater risk of ALS [2], although in other populations, no association between VEGF polymorphisms and ALS is seen [2,10,11]. This does not exclude the possibility that dysregulation of the VEGF signalling pathway plays a role in the pathogenesis of ALS in these patients, as genetic factors may also control response to VEGF [12]. We have previously shown that expression of VEGFR2 is downregulated in the CNS of ALS patients [4]. In this study we have addressed the question of whether sequence variation in the regulatory regions of the VEGFR2 gene in ALS patients may contribute to lower expression of this VEGF agonist receptor. We have sequenced important functional regions of this candidate gene, to determine whether sequence variation in the coding regions could affect function of the receptor, and therefore responsiveness to VEGF in ALS.

No mutations or differences in polymorphism frequency were identified in the ALS population, in the regulatory regions or 4 coding regions of probable functional significance. These findings do not support the hypothesis that genetic factors reduce VEGF responsiveness in ALS, via downregulation of expression or function of VEGFR2. This study had a power of 89% to detect a relative risk of 1.8 at 0.05 significance level, therefore the lack of association between VEGFR2 promoter region polymorphisms and ALS is unlikely to be due to sample size.

Mechanisms other than genetic factors may reduce VEGFR2 expression in ALS. In ALS patients without 'at-risk' VEGF haplotypes, decreased plasma levels of VEGF were also identified [2]. Similarly, reduced expression of VEGFR2 may occur secondary to other disease mechanisms, such as gene downregulation due to dysregulation of transcription factors, which is seen in a mutant SOD1 cell model of ALS [13]. VEGFR2 is a large gene with 30 exons, and although we selected functionally significant regions of the gene, we have not excluded the possibility that sequence variation in other exonic regions could disrupt the function of this protein in ALS patients.

## Conclusion

There is no evidence from sequencing of the regulatory regions and 4 functionally significant exonic regions of the VEGFR2 that genetic variation in this agonist receptor causes downregulation of VEGF signalling in ALS patients.

## Abbreviations

VEGF Vascular endothelial growth factor

VEGFR2 Vascular endothelial growth factor receptor 2

ALS Amyotrophic lateral sclerosis

SOD1 Superoxide dismutase 1

PMA Progressive muscular atrophy

PLS Primary lateral sclerosis

PBP Progressive bulbar palsy

UTR Untranslated region

## Competing interests

The author(s) declare that they have no competing interests.

## Authors' contributions

AB carried out molecular genetic studies, sequence alignment and drafted the manuscript

BW carried out molecular genetic studies and sequence alignment

HN collected DNA samples and managed patient databases

JH carried out DNA extraction and managed patient databases

PS conceived of the study and helped to draft the manuscript

All authors read and approved the final draft of the manuscript.

## Pre-publication history

The pre-publication history for this paper can be accessed here:


